# Review of the Developments of Bacterial Medium-Chain-Length Polyhydroxyalkanoates (mcl-PHAs)

**DOI:** 10.3390/bioengineering9050225

**Published:** 2022-05-21

**Authors:** V. Uttej Nandan Reddy, S. V. Ramanaiah, M. Venkateswar Reddy, Young-Cheol Chang

**Affiliations:** 1 Department of Biotechnology, Koneru Lakshmaiah Education Foundation, Guntur 522502, Andhra Pradesh, India; vemireddyuttej@gmail.com; 2 Food and Biotechnology Research Lab, South Ural State University (NRU), 76, Lenin prospekt, Chelyabinsk 454080, Russia; ramanasudarsan@gmail.com; 3 Center for Biotechnology and Interdisciplinary Studies, Rensselaer Polytechnic Institute, 110 8th Street, Troy, NY 12180, USA; motakv@rpi.edu; 4 Course of Chemical and Biological Engineering, Muroran Institute of Technology, Hokkaido 050-8585, Japan

**Keywords:** polyhydroxyalkanoates, mcl-PHAs, scl-PHAs

## Abstract

Synthetic plastics derived from fossil fuels—such as polyethylene, polypropylene, polyvinyl chloride, and polystyrene—are non-degradable. A large amount of plastic waste enters landfills and pollutes the environment. Hence, there is an urgent need to produce biodegradable plastics such as polyhydroxyalkanoates (PHAs). PHAs have garnered increasing interest as replaceable materials to conventional plastics due to their broad applicability in various purposes such as food packaging, agriculture, tissue-engineering scaffolds, and drug delivery. Based on the chain length of 3-hydroxyalkanoate repeat units, there are three types PHAs, i.e., short-chain-length (scl-PHAs, 4 to 5 carbon atoms), medium-chain-length (mcl-PHAs, 6 to 14 carbon atoms), and long-chain-length (lcl-PHAs, more than 14 carbon atoms). Previous reviews discussed the recent developments in scl-PHAs, but there are limited reviews specifically focused on the developments of mcl-PHAs. Hence, this review focused on the mcl-PHA production, using various carbon (organic/inorganic) sources and at different operation modes (continuous, batch, fed-batch, and high-cell density). This review also focused on recent developments on extraction methods of mcl-PHAs (solvent, non-solvent, enzymatic, ultrasound); physical/thermal properties (Mw, Mn, PDI, Tm, Tg, and crystallinity); applications in various fields; and their production at pilot and industrial scales in Asia, Europe, North America, and South America.

## 1. Introduction

**Figure 1:**
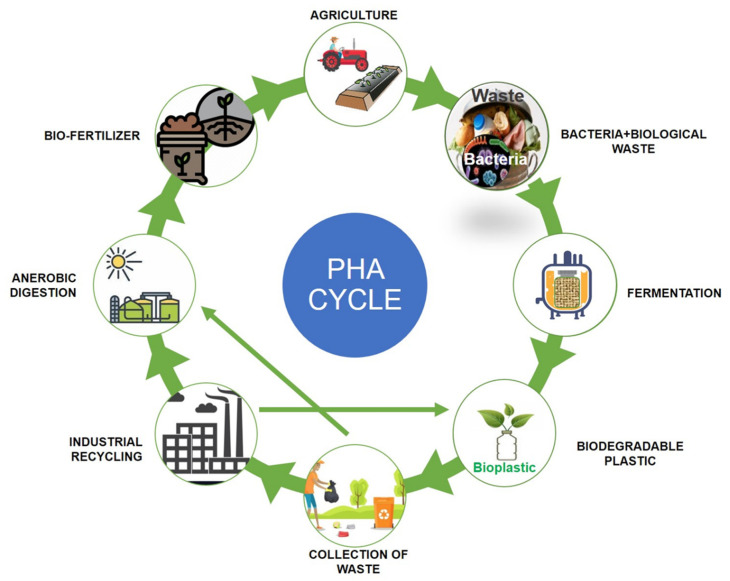
Polyhydroxyalkanoates (PHA) production and degradation cycle.

Polyhydroxyalkanoates (PHAs) are a type of biopolymer developed as intracellular carbon/energy storage materials which have a wide range of material characteristics. PHAs are identified as granular inclusion bodies after extraction from cells, these are becoming popular as prospective replacements for traditional plastics in various applications, including food packaging industries, cultivational fields, scaffold preparation, and biomaterial implants [1,2]. PHAs are classified based on the length of the 3-hydroxyalkanoate (3HA) repeat units, the repeat units of PHAs with short chain length (scl-PHAs) are generally 4–5 carbon atoms long (e.g., 3-hydroxybutyrate, 3HB and 3-hydroxyvalerate, 3HV units), medium chain length (mcl-PHAs) are 6–14 carbon atoms long (e.g., 3-hydroxyhexanoate, 3HHx, 3-hydroxyheptanoate, 3HHo), and long chain length (lcl-PHA) are more than 14 carbon atoms (e.g., 3-hydroxyhexadecanoate) [3,4]. Rakkan et al. (2022) reported the production of mcl-co-lcl PHAs (72 to 75% of DCW) from *Enterobacter* sp. strains TS3 and TS1L using glucose [5]. Mcl-PHAs are soft, elastomeric, and have less crystallinity, a lower melting point, and lower glass transition temperature [6]. Nutrient limiting conditions are required for PHA generation in *Pseudomonas oleovorans*, *Pseudomonas putida*, and *Ralstonia eutropha*, but not in recombinant *Escherichia coli* and *Alcaligenes latus* [7]. High manufacturing costs are a key impediment to the commercialization of PHAs. Carbon conversion yield (g/g), titer or volumetric yield (g/L), and productivity (g/L/h) are crucial in this scenario [8]. Aside from production criteria, low-cost downstream processing techniques and PHA manufacturing that fulfils cost performance standards have remained difficult to achieve. This has prompted researchers to focus on improving PHA fermentation and downstream processing efficiency to lower total costs [9,10]. The PHA production and degradation cycle is explained in Figure 1.

A significant aim for PHA synthesis is to obtain high yields in high cell-density cultures (HCDCs). Bioethanol and yeast that produces single-cell proteins were used to construct HCDC in the beginning. Because of its benefits, such as reduced culture volume and residual liquid, cheaper production costs, and lower capital investment, HCDC technique is favored over low cell density technologies [11,12]. Dry cell weight (DCW) above 100 g/L is termed HCDC for PHA synthesis, a DCW above 50 g/L is regarded high for recombinant protein production [11,13,14]. More details on PHA production through HCDC were provided in Section 3.2. The species of *Bacillus* and *C. necator* are generally known to produce scl-PHAs, whilst *Pseudomonas* species known to produce mcl-PHAs. PHAs including Scl and Mcl can be developed by the species of *Alcaligenes* and *Rhodococcus*. PHAs have been used to construct a variety of drug carriers, cardiac patches, vascular grafts, nerve conduits, heart valves, artificial blood vessels, subcutaneous implants, orthopaedical pins, stents, wound dressings, and slings [15]. In addition, the FDA authorized P4HB for medical usage as absorbable sutures in the year 2007 [16]. Poly-3-hydroxyhexanoate (P3HO) has been used for soft (cardiovascular and neurologic) and hard (bone) tissue engineering [17,18]. By lowering mitochondrial damage, methyl esters of 3HB were used as drugs to treat Alzheimer’s disease [19]. Memory enhancers have also been shown to be sodium salts of 3HB monomers [20].

Unlike other polymers used before, which degrade through bulk degradation, PHAs degrade through controlled surface erosion [15]. Controlled degradation must be used to preserve implant integrity in vivo. PHAs have been thoroughly tested both inside and outside of the labs to confirm that they are biodegradable. PHAs also degrade far slower than polylactic acid (PLA), making them suited for long-term applications. This review article focused on mcl-PHA production using organic/inorganic carbon sources and various types of the fermentation strategies. We also discussed recent developments on cost-effective downstream processing methods, thermal/mechanical properties of mcl-PHAs, applications of mcl-PHAs, and their worldwide production at pilot and industrial scale.

## 2. Mcl-PHA production

### 2.1. Inorganic Carbon Sources

**Figure 2:**
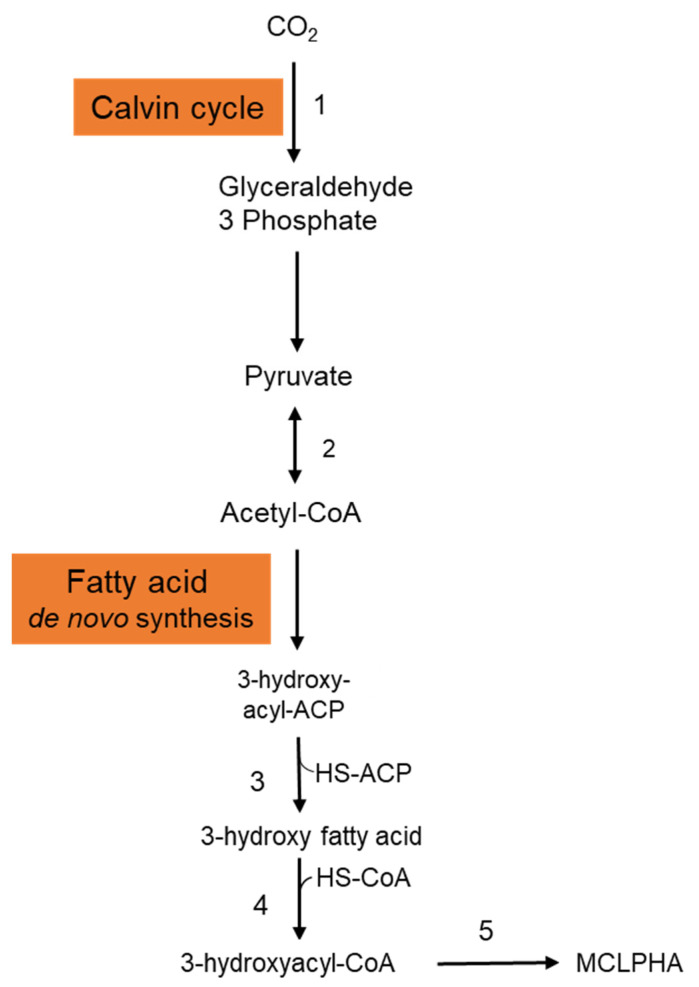
Metabolic pathway involved in the synthesis of mcl-PHA from CO_2_ in *Rhodospirillum rubrum*. This figure was generated with the information from [21]. 1: Ribulose 1,5-bisphosphate carboxylase; 2: Pyruvate synthase; 3: 3-hydroxyacyl-ACP thioesterase; 4: MCL fatty acid CoA ligase; 5: PHA synthase.

It is crucial to employ natural substrates for microbial PHA synthesis to reduce manufacturing costs. The ideal feedstock is one that does not compete with human food. Syngas is a carbon monoxide and hydrogen mixture that may be produced by pyrolyzing organic waste. PHAs may be synthesized by a variety of microorganisms using syngas. Heinrich et al. (2016) genetically engineered the *Rhodospirillum rubrum* S1 to create a heteropolymer of 3-hydroxydecanoic acid and 3-hydroxyoctanoic acid [P(3HD-co-3HO)] from artificial syngas including CO and CO_2_ [21]. Three genes—3-hydroxyacyl-ACP thioesterase (*phaG*), mcl-fatty acid CoA ligase (PP 0763), and a PHA synthase (*phaC1*)—from *P. putida* KT2440 were chosen for overexpression. To investigate the impact of syngas-mediated gene overexpression, the CO-inducible PcooF promoter was employed. A recombinant mutant produced P(3HD-co-3HO) up to 7.1% (wt/wt) of the DCW. Furthermore, enhanced mcl-PHA synthesis and increased gene expression via the PcooF promoter resulted in a greater molar fraction of 3HO in the generated copolymer than the Plac promoter, which regulated expression on the original vector. The polymer has a molecular mass of 124.3 kDa, melting point of 49.6 °C, and glass transition temperature of 41.1 °C. According to GC analysis, the polymer-isolate fractions were 55.6 mol % 3HD, 44.2 mol % 3HO, and less than 0.2 mol % 3HH. The partial disintegration of the accumulated polymer might be connected to the activity of a lipase identified in *R. rubrum*, as these enzymes have been shown to digest PHAs with varying chain lengths [22]. Figure 2 depicts the metabolic pathway carried in the production of mcl-PHAs from CO_2_ in recombinant *R. rubrum*. After entering into the cytoplasmic membrane, CO_2_ is fixed via ribulose 1,5-bisphosphate carboxylase through the Calvin cycle and generates glyceraldehyde-3-phosphate, which is subsequently converted to the pyruvate. Pyruvate is further converted into acetyl-CoA by the action of the enzyme pyruvate synthase. Acetyl-CoA is then entered into the fatty acid de novo synthesis cycle and generates 3-hydroxyacyl-ACP. The thiolysis of 3-hydroxyacyl-ACP is catalyzed by the 3-hydroxyacyl-ACP thioesterase and produces 3-hydroxy fatty acid. Activation of 3-hydroxy fatty acids occurred by the addition of CoA, which is catalyzed by the enzyme mcl-fatty acid CoA ligase. The enzyme PHA synthase polymerizes the 3-hydroxyacyl-CoA into mcl-PHA [21].

PHAs were produced using *C. eutrophus* B-10646 with scl-mcl monomer units [23,24]. The cells were grown on a mineral medium with a main growth substrate (gas) comprising a 1:2:7 ratio of CO_2_: O_2_: H_2_ (by volume). The gas mixture was continuously pushed through the culture at a rate of 10–12 L/min in a 10 L bioreactor, the oxygen volume coefficient of mass transfer was 460 h^−1^ (kLa). By varying the butyrolactone concentration (3–5 g/L), copolymers with varying molar fractions of 3HB/4HB (10.0 to 51.3%), 3HV (0.3–0.5%), and 3HHx (0–0.4%) were obtained. Tanaka et al. (2021) used recombinant *C. necator* strains and a mineral salts medium [25]. A sterile filter was employed to introduce a substrate gas mixture with a ratio of H_2_/O_2_/CO_2_ = 8:1:1. The quantity of PHAs were determined by gas chromatography analysis. In all the cultures investigated, the PHAs generated are a copolyester of 3HB and 3HHx (47.7%). Löwe et al. (2017) used synthetic bacterial co-culture to generate mcl-PHAs (C6, C8, C10, and C12) from CO_2_ [26]. *P. putida* cscAB fixes CO_2_ and changes it to sucrose, and then transfers into the culture filtrate, this sugar serves as a carbon source for *Synechococcus elongatus* cscB, which converts it to PHAs that accumulate in the cytoplasm. Using a nitrogen-limited method, they were able to achieve a maximum PHA production rate of 23.8 mg/L/day and a maximum titer of 156 mg/L.

### 2.2. Organic Carbon Sources

Mcl-PHA production from various organic carbon sources were summarized in Table 1.

#### 2.2.1. Fatty Acids

*R. eutropha* grown on animal fat waste produced 45 g/L biomass which contains 60% PHA [37]. In the *R. eutropha* recombinant strain, the *R. aetherivorans* has expressed I24 PHA synthase gene, and PHA with HHx units is produced by *P. aeruginosa* hydratase gene (*phaJ*) [27]. The total biomass produced was 139 g/L with 74% of co-polymer, P (3HB-co-19 mol % 3HHx). Sato et al. observed that, in utilizing butyrate and palm kernel oil as carbon and energy sources, recombinant *C. necator* H16 produced good amount of P (3HB-co-19 mol % 3HHx). Furthermore, the scientists demonstrated that the 3-HHx% in KNK005 phaA-deactivated mutant strains is boosted by the butyrate. This culture environment produces higher biomass (171 g/L) and HHx copolymer in the cells (81%) with PHA titers of 139 g/L [38]. Cai et al. (2009) produced a *P. putida* KTMQ01 recombinant. The recombinant’s DCW was 86% mcl-PHA [39]. Genetically altered *P. putida* KT2440 employs inexpensive substrates, such as xylose and octanoic acid, to produce mcl-PHA at a low cost. The cells accumulated mcl-PHA to a level of 20% [40].

A PHA-negative mutant of *R. eutropha* was created using the *phaC* gene from *Aeromonas caviae*, produced 3HB copolymer comprising 5 mol % 3HHx using soybean oil (20 g/L) in fed-batch mode [41]. DCW and % PHA were 133 g/L, 72.5% respectively. *A. hydrophila* 4AK4 produced P(3HB-co-3HHx), which contains 15% HHx from dodecanoate [42]. The co-expression of *phaC* with *phaP* and *phaJ* resulted in a 3HHx level of 34 mol % in the copolymer. When dodecanoate was used as the only carbon source, 54 g/L DCW and 52.7% PHA were observed [43]. The wild-type strain produced 40.4 g/L of DCW, and 54.6% of P(3HB-co-3HHx) [44]. This modified strain used plant oils successfully, produced 88.3 g/L dry biomass with a polymer content of 57% (3HB-co-3HHx) [45]. Tufail et al. investigated the use of leftover braising liquid as a substrate for PHA production using *P. aeruginosa* (KF270353) for 72 h. PHAs (53.2%) and DCW (23.7 g/L) were detected in the highest amounts in waste braising liquid [46]. Ruiz et al. (2019) produced 159.4 g/L of DCW, and 57 g/L of mcl-PHA using fatty acids generated from waste cooking oil as the carbon source, and *P. putida* KT2440 as biocatalyst [28].

#### 2.2.2. Carbohydrates

**Figure 3:**
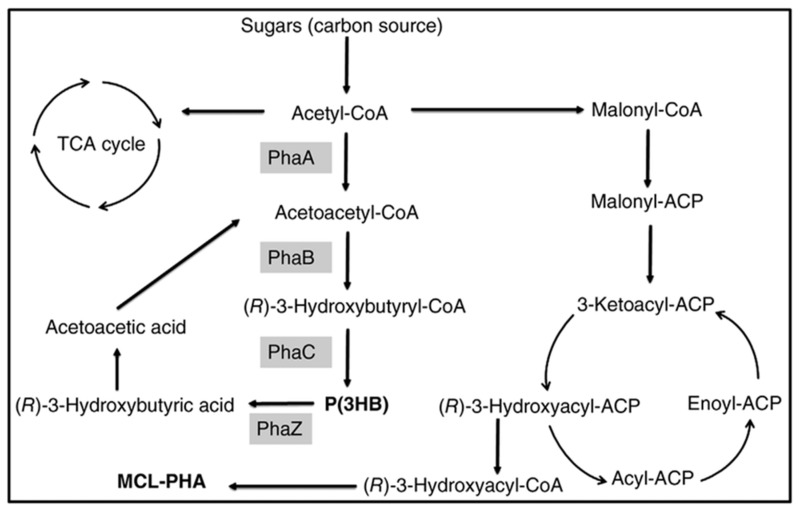
Metabolic pathway involved in the synthesis of PHAs from sugars. PhaA: β-ketothiolase; PhaB: β-ketoacyl-CoA reductase; PhaC: PHA synthase; PhaZ: PHA depolymerase.

P(3HB-co-3HHx) with 14 mol % HHx units was produced with gluconate using recombinant *A. hydrophila* 4AK4 strain [47]. Poblete-Castro et al. (2014) produced PHA using *gad* (gluconate dehydrogenase) deleted mutant, *P. putida* KT2440 [48] under fed-batch mode. The DO-stat feeding method generated the 67% of mcl-PHA with 62 g/L DCW [46]. Fed-batch cultivation of *P. putida* KT2440 on mixed substrates of acrylic acid, nonanoic acid, and glucose (0.05: 1.25: 1) generated 71 g/L of DCW with 75% of PHA which contains the 89 mol % 3HN [29]. However, the concentration of 3HN was decreased to 65 mol % in the absence of acrylic acid. The amount of mcl-PHA produced increased 10-fold when dodecanoic acid was utilized as the feedstock by Zhao et al. (2020) [49]. Liu et al. (2019) reported that *Pseudomonas-Saccharomyces* produced up to 152.3 mg/L mcl-PHA using xylose as a carbon root [50]. The inclusion of *S. cerevisiae* in the consortium produced cell mass sedimentation. Sugarcane biorefinery derived sucrose hydrolysate and decanoic acid used in fed-batch cell cultivations produced 33% PHA in 53.4 g/L of DCW using *P. putida* KT2440 [51]. A β-oxidation metabolism in the *R. eutropha* mutant was studied for P(3HB-co-3-HHx) development from soybean oil [52]. The removal of *fadB1* (enoyl-CoA hydratase/3HACoA dehydrogenase) in *R. eutropha* recombinant strains with additional genes generating (R)-enoyl-CoA hydratases resulted in a 6–21% 3-HHx content in the copolymer [52]. Figure 3 depicts how the metabolic pathway operates in the production of mcl-PHAs from sugars.

#### 2.2.3. Organic Residues/Wastes and Others

**Figure 4:**
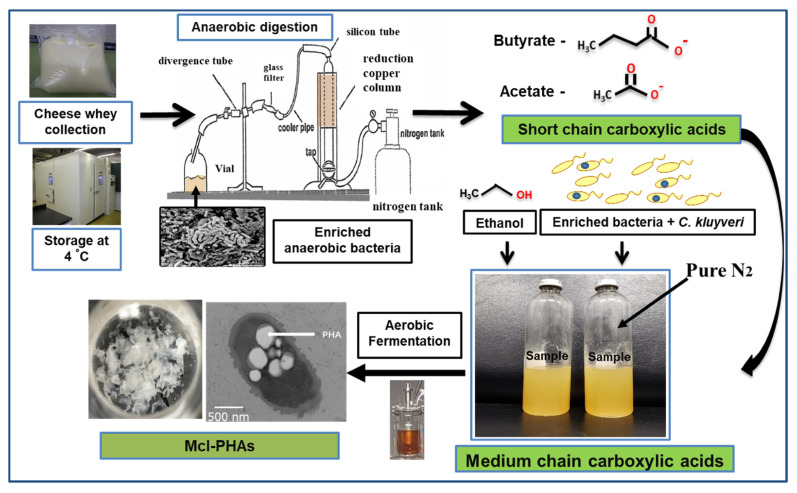
Hypothetical flow chart describing steps involved in mcl-PHA production from cheese whey.

PHA production was carried out using organic acids/or sugar-based compounds derived from renewable waste materials [53]. Davis et al. (2013) produced mcl-PHA (25–34%) from perennial ryegrass biomass using *P. fluorescens* 555 and *P. putida* W619 [54]. Awasthi et al. (2021) reported about the mcl-PHA production using watermelon waste residues [55]. Muhr et al. (2013 a,b) produced mcl-PHA (22–30% DCW) from *P. chlororaphis* DSM 50083 and animal-derived waste [6,56]. Grape pulp was used to produce mcl-PHA from *P. resinovorans* in a 3.7 L bioreactor [57]. Chicken feathers were used to produce mcl-PHA with *P. putida* KT2440, the polymer consisted of 3-HHx (27.2 mol %) and 3-HHo (72.8 mol %) monomer units [58]. Apple pomace, the residue which is left out after processing of apple serves as a potential carbon source to produce PHAs [59]. Blanco et al. (2021) reviewed the PHA production using various substrates [60]. Apart from the above-mentioned carbon sources, there is a scope to produce mcl-PHAs from waste materials such as food waste, molasses, lingo-cellulosic biomass, cannery waste, biodiesel industry waste, paper-mill wastewater, coffee waste, and cheese whey [61]. As an example, production from cheese whey shown in Figure 4. PHAs were produced using synthetic substrates, waste materials, and toxic compounds [62,63,64,65,66,67,68,69,70,71,72].

#### 2.2.4. Vegetable Oils

Coconut oil containing both medium (C6–C14) and long (>C14) chain length fatty acids, lauric acid (C12:0) and myristic acid (C14:0) (up to 55%) are the main fatty acids [73]. *Pseudomonas* sp. is recognized to produce PHAs through fatty acids. Fatty acids are shortened by basically 2, 4, or 6 carbon atoms after each cycle of β-oxidation. In nature, *Pseudomonas* sp. is known for its adaptability, and it can make mcl-PHAs from a range of carbon feedstocks. PHAs (58% DCW) were produced in 20 L bioreactor in a batch mode by *P. mendocina* CH50 (NCIMB 10541) using coconut oil at a concentration of 20 g/L [74]. The optical density increased to 31 after 48 h, and the pH decreased slightly during fermentation, nitrogen content declined from 0.12 g/L to 0.015 g/L shows that the fermentation took place in a nitrogen-limiting environment. The mcl-PHAs terpolymer was composed of 3HO-3HD-3HDD. P(3HO-3HD-3HDD) has a molecular weight of 333 kDa and a PDI (polydispersity index) of 2.37, whereas mcl-PHAs with both unsaturated and saturated groups have an average molecular weight of 60–410 kDa [75]. *Pseudomonas* sp. Gl01 was used to produce mcl-PHA using palm oil and waste rapeseed oil. The purified polymers consisted of monomers ranging from C6 to C16 [76,77]. Song et al. (2008) used *Pseudomonas* sp. strain DR2 and the waste vegetable oil to produce mcl-PHA in the range of 24–38% [78]. Previous studies find out the importance of C/N balance for PHA production [79,80,81]. Carbon and nitrogen concentrations play a major role in PHA production. Mohan and Reddy (2013) applied design of experimental methodology using Taguchi orthogonal array to evaluate the influence and specific function of eight important factors and mentioned that glucose at 6 g/L and NH_4_Cl at 100 mg/L concentrations are best for higher PHA accumulation [80]. Reddy et al. (2015) reported that *phaC* gene expression was 5.37 folds higher at 100 mg/l nitrogen concentration than other concentrations [63]. The precursor molecules required for mcl-PHA synthesis in bacteria are generated through three different metabolic routes, which depend on the type of carbon source that existed in the medium. If carbohydrates are the main carbon source, the de novo fatty acid pathway is dominant, and fatty acids are the main carbon source, the β-oxidation pathway is dominant. The third pathway is chain elongation, and this pathway utilizes the precursors produced from both carbohydrates (acetyl-CoA) and fatty acids (acyl-CoA). All the three metabolic pathways generate different intermediate precursors—such as (R)-3-hydroxyacyl-acyl carrier protein, 2-trans-enoyl-CoA, (S)-3-hydroxyacyl-coA, and 3-ketoacyl-CoA—which are involved in the mcl-PHA synthesis. The hypothesis is that the (S)-3-hydroxyacyl-CoA, and 3-ketoacyl-CoA are subsequently converted to (R)-3-hydroxyacyl-CoA by the action of enzymes, 3-hydroxyacyl-coA epimerase, and 3-ketoacyl-ACP reductase respectively. The enzyme, (R)-specific enoyl-CoA hydratase (*PhaJ*)—which catalyzes the 2-trans-enoyl-CoA to (R)-3-hydroxyacyl-coA—plays a critical role in supplying monomer units from β-oxidation to PHA synthesis. (R)-3-hydroxyacyl-ACP-CoA transferase (*PhaG*), which has been identified in *P. putida* and *P. aeruginosa* plays, an important role in the metabolic connection of de novo fatty acid biosynthesis with mcl-PHA synthesis. PhaG catalyzes the conversion of (R)-3-hydroxyacyl-ACP to (R)-3-hydroxyacyl-CoA and contribute to mcl-PHA synthesis from gluconate or other carbohydrate sources. In the final step of mcl-PHA synthesis, the enzyme PHA synthase (*PhaC*) catalyzes the conversion of (R)-3-hydroxyacyl-CoA molecules into mcl-PHA [6,28,40,48,52,54,56].

## 3. Mcl-PHA Production at Various Modes of Operations

Mcl-PHA yields are optimized in batch, fed-batch, and continuous operation methods (Table 2). Sugar has been the most common carbon source used by most industries to manufacture PHAs during the last 20 years. Sugar can be produced from several biomass sources such as sugarcane, beet, molasses, and bagasse. They are plentiful and simple to obtain, and bacteria can swiftly digest and convert them to PHAs. Large PHA enterprises have selected this strategy due to the ample availability of raw materials and the ease of operation.

### 3.1. Batch Mode

In batch mode, carbon/nitrogen sources will be added into the reactor at initial hours of incubation, and no extra nutrients will be introduced afterwards [90]. Batch fermentation procedures, in general, lead to a lower PHA yields. This is due to the breakdown of PHAs that produced in the cultivation process [91]. Under batch conditions, Rai et al. (2011) achieved a DCW of 0.8 g/L which contains 31% homopolymer, P3HO using *P. mendocina* [82].

### 3.2. Continuous Mode

This mode maintains a constant growth rate of microbes under ideal conditions. Consequently, by continuing to cultivate at high specific growth rates, production may be enhanced. Continuous culture is also beneficial since it reduces the need for bioreactor shutdowns and cleaning. Furthermore, the continuous culture prevents washout even at high dilution rates; therefore, product concentration and production may rise. The continuous culture is maintained by new media to the reactor, which feeds the cells with new nutrients. Products and effluent are continuously removed to maintain a consistent bioreactor workload. Jung et al. used *P. oleovorans* to derive mcl-PHAs from n-octane in a two-step continuous process. With two-stage fermentation, one can focus on accumulating PHAs in one reactor while accumulating biomass in another. Under these conditions, 18 g/L DCW, 63% of PHAs were achieved [83]. Egli et al. (1991) employed chemostat cultivation atmosphere to produce PHAs from *P. putida* GPo1 [92]. According to this study, PHAs may form if nitrogen limitation is applied [93].

### 3.3. Fed-Batch Mode

In the fed-batch method, cells proliferate until the desired cell density is attained with a steady supply of carbon sources and necessary nutrients. The feeding of nutrients and carbon sources maintains a consistent rate of growth, limiting the formation of by-products. There are two different types of feed-batch operations, one is the development of growth-related products, and the other one is product formation that occurs only under the non-growth associated conditions. PHA development usually occurs in two stages. First, the log stage is performed so that the cells gained the sufficient biomass. The second stage of polymer synthesis entails feeding all the essential materials into the bioreactor [94]. The second stage usually occurs when an essential nutrient, such as nitrogen, phosphorous, and oxygen limited conditions.

Most of the mcl-PHA production studies were conducted in a batch-fed mode. The pH and DO stats are utilized during the fermentation process to keep the pH and DO at specific levels. Lee et al. (2000) got 51% of PHAs, 141 g/L of DCW, using *P. putida* and oleic acid [84]. Davis et al. (2015) employed *P. putida* KT2440 in a two-stage fed-batch mode with glucose and nonanoic acid [13]. Cells were fed glucose during the biomass developing phase, while nonanoic acid was provided during the PHAs developmental phase and obtained 32% of PHAs, 102 g/L of DCW [13]. An oxygen-limited fed-batch growth approach with *P. putida* LS46 and octanoic acid as a substrate in a 7 L bioreactor achieved 29 g/L of DCW, 61% of PHA [30]. Octanoic acid toxicity in *P. putida* LS46 cells might explain the low biomass accumulation. Gao et al. (2016) created mcl-PHA using a mixture of decanoic and acetic acids in a fed-batch culture of *P. putida* KT2440 [85]. Acetic acid used to inhibit the crystallization of decanoic acid. Different glucose/acetic acid/decanoic acid ratios were used to find co-feed ratios that resulted in higher mcl-PHA yields. At the ideal ratio (4:1:5), 75 g/L of total DCW and 74% of PHA content were attained. Sun et al. (2009) demonstrated that *P. putida* KT2440 developed mcl-PHA by co-feeding glucose and nonanoic acid [31]. After exponential, as well as subsequent constant feed, with 1:1 (*w*/*w*) nonanoic acid: glucose, 71 g/L of total DCW and 56% of PHA were observed. Cerrone et al. (2014) demonstrated that the *P. putida* CA-3 HCDC was formed in a two-stage fermentation using decanoic and butyric acid [86]. To boost the maximal yield of mcl-PHA, the isolates were cultivated first on butyric acid (biomass growth phase) and then on a combination of butyric and decanoic acid (20:80 *v*/*v* ratio) during the PHA synthesis stage. This approach resulted in 71.3 g/L of DCW with 65% of PHA [86].

Sun et al. (2007) used the *P. putida* KT2440 to manufacture mcl-PHA from nonanoic acid, obtained 70 g/L of DCW with 75% PHAs [87]. Diniz et al. (2004) employed *P. putida* IPT 046 to explore various feeding patterns such as exponential preceded by constant feed for production of mcl-PHA [32]. The exponential feeding method yields a maximum 40 g/L DCW and a 21% of PHAs. However, when phosphate was limited, 50 g/L of DCW with 63% PHAs was obtained [32]. *P. oleovorans* ATCC 29347 was cultivated under pH-stat fed-batch conditions with octanoic acid as the carbon source and reported 63 g/L of DCW with 62% PHAs [88]. Kim et al. (1997) investigated a two-stage fed-batch approach using *P. putida* BM01 [89]. Glucose and octanoate were supplied in growth and PHAs development stages. This approach yielded 55 g/L of DCW with 66% of PHA. Higher DCW (125.6 g/L) with 55% PHAs observed using *P. putida* KT2440 [95].

## 4. Industries Producing PHAs

Scl-PHA are produced by various industries; however, very few industries produce mcl-PHAs. In this section, we provide information regarding industries that produce PHAs in various regions.

### 4.1. Europe

In 2007, Biomer Biotechnology Co. (Starnberg, Germany) produced 10 tonnes of Biomer bio polyester. Biomer’s objective has been to integrate with agricultural food industries, which they believe will offer the technology needed to create PHAs from its waste [96]. Bio-on founded LUX-ON with the purpose of generating PHAs using CO_2_ as organic load. The system also gathers sustainable solar energy to power the bioproduction process. Sugar cane molasses, sugar cane, food wastes, waste cooking oil, glycerol, and carbohydrates are among the feedstocks utilized [97]. Paques, a Dutch company, employs natural bacteria and methods to generate biodegradable PHBV biopolymers from waste streams [98]. Bioextrax is a Sweden-based company producing PHAs using bacteria from sucrose [99]. BASF is a German-based company producing biopolymer, Ecovio^®^ with special material properties such as flexibility and toughness. Guzik (2021) summarized the recent developments occurred in Jerzy Haber Institute of Catalysis and Surface Chemistry of the Polish Academy of Sciences in collaboration with other groups [100].

### 4.2. Asia

Tianan Biologic [101] is a biopolymer specialist in the production and use of PHBV and PHB. To make the copolymer, *C. necator* is fermented with D-glucose and propionic acid. The fermentation facilities are in the city of Ningbo, China. Since its founding in 2000, the company has generally been recognized as the largest maker of PHBV, with an annual capacity of 2000 metric tons. In 2004, they were the first company in the world to commercially synthesize PHBV using water-based extraction technology. The extraction method has been patent-protected. Tianjin GreenBio Material Co. is China’s first company to produce 10,000 tonnes of PHAs per year. They have developed completely biodegradable granules for the manufacturing of blown film (SoGreen 2013). They also developed PHAs foam pellets, which can be converted into completely biodegradable foams for usage in food service and appliance packaging. The PHAs are created using a P(3HB-co-4HB) copolymer.

Mitsubishi (Tokyo, Japan) manufactures P(3HB) at both research and pilot scales under the brand name Biogreen^®^ [102]. Kaneka Corporation (Tokyo, Japan) manufactures P(3HB-co-3HHx) under the brand names Kaneka PHBH^®^ and AONILEX^®^, with an annual capacity of 100 tonnes. To make it, plant oils and fatty acids are used as the primary raw material in a microbial fermentation process [103]. Metabolix, located in Massachusetts, has completed the sale of its PHA biopolymer intellectual property to an associate company, CJ Cheil Jedang Co (Seoul, Korea) for 10 million USD. The acquisition includes patents on production and application, as well as microbes used in Metabolix’s manufacturing procedures [104].

### 4.3. North and South America

PHAs are being commercialized for high-value biological applications by several PHA manufacturers. These firms include Terra Verdae Bioworks (Edmonton, AB, Canada), PolyFerm (Kingston, ON, Canada), and Tepha Inc. (Lexington, MA, USA). Among the products provided are heart valves, scaffolds, ecological sutures, and materials for regulated distribution. These product categories have lower volume but higher profit margins. Polyferm Canada [105] produces mcl-PHAs under the brand name. Versa MerTM using naturally selected microorganisms and basic materials such as vegetable oils and sugars. The applications they are now working on include medical devices, sealants, adhesives, and polymer modifiers. Danimer Scientific manufactures produces PHAs under the trade name NodaxTM in the United States (Bainbridge, GA, USA). They entered the PHAs industry in 2007 after receiving knowledge from Procter & Gamble (Cincinnati, OH, USA). Danimer created a copolymer with a 3HB unit and a mcl-PHA repeat unit [106]. NodaxTM PHAs are manufactured from easily accessible feedstock and are completely renewable. Mango Materials, a company based in the United States, is producing PHAs from methane.

PHB Industrial S/A is a Brazil based company that manufactures and sells Biocycle^®^ disposable polymers, PHB, and P (3HB-co-3HV) since September 2000 [107]. Until 2015, the firm functioned on a small scale and sold its commodities to Japan. Metabolix, Inc. released Yield10 in 2015, and it was listed on NASDAQ. PHB and its copolymers are manufactured by Yield10 Bioscience under the MillerTM brand [108]. MillerTM was marketed by Telles, a joint venture between Archer Daniels Midland Firm and Metabolix. TephaFLEX^®^ produces P4HB. After detoxification, P4HB is converted into healthcare products such as films, sutures, and fabrics [109]. P4HB is digested to yield 4HB, a naturally occurring element of the vertebrate body. Tepha’s TephELAST is more elastic than TephaFLEX and has been employed in the production of medical devices. These polymers are created using pilot and research platforms. Newlight Technologies developed a unique biocatalyst to produce PHAs from CO_2_ and biogas using microorganisms obtained from the Pacific Ocean. Newlight created the AirCarbon polymer by combining methane from a California cattle farm with air [110,111].

## 5. News on PHAs

The PHAs market is predicted to reach 121 million USD by 2025, according to *Markets and Markets* [112]. The rising demand for PHAs in various industries—such as packaging, biomedical, and agricultural—is driving market expansion. Several factors will propel the PHAs sector, including public awareness of the depletion of hydrocarbons and the development of sustainable, eco-friendly bioplastics. Europe is the world’s largest PHAs market in terms of volume and value, followed by North America and Asia. PHAs are more expensive than standard polymers, which is one of the key impediments to the market’s growth. Biodegradable polymers, such as PHAs, have higher production costs than normal plastics, ranging from 20% to 80% more. This is mostly due to the high polymerization cost of biodegradable polymers, which is because most of the technologies are still in the research stage. These bio-based manufacturing methods and materials are still in the early stages of development and have not yet achieved the level of commercialization that their petroleum-based equivalents have.

The company Mars plans to reduce virgin plastic use by 25% by the year 2025 and make all plastic packaging reusable, recyclable, or biodegradable [113]. Mars is aiming for 100% biodegradable, recyclable, or biological plastic packaging by 2025, as well as a 25% reduction in virgin plastic consumption, a 30% average recycled content in plastic packaging, and recycling requirements for customers in all important markets. Maltesers replaced the plastic interior of the candy box with a water-based coating, reducing 82 metric tons of plastic waste. The first-ever Mars Wrigley Gum bottle with 30% recycled content is now available on the German market. This is an industry-leading move, reducing unused plastic use by approximately 350 tons annually. Orbit Megapack has released an on-pack recycling guide on How2Recycle, which provides a step-by-step guide on whether and how to recycle each part of the gum pack. Balisto has partnered with German retailer EDEKA Minden-Hannover to offer the first chocolate bar with paper-based packaging, reducing packaging plastic use by approximately 440 kg. M&M’s launched recyclable packaging in the M&M’s Choco 300 g pouch in France. Mars Wrigley partnered with Danimer Scientific, a leading developer and manufacturer of biodegradable materials, to develop an innovative home compostable packaging for its products. Colgate designed recyclable toothpaste tubes so they can go into curbside recycling bins. This could eventually keep a billion tubes out of landfills each year [114].

## 6. Extraction of PHAs

In terms of product quality, price, and environmental effect, downstream processing is a significant stage in PHAs manufacture. Following fermentation, there are two fundamental ways for recovering PHAs: (i) dissolve the biomass in acid, alkaline solutions, detergents, protease and extracting the pellets; or (ii) direct solvent separation of PHAs from the bacteria [115]. Enzymatic degradation of non-PHA biomass is one solvent-free approach for releasing PHA granules from cells. Certain proteins have been found on the membrane of PHA granules, such proteins include synthases, PHA depolymerize enzymes, polymer membrane expressed protein (e.g., phasins), and other proteins that influence pellet organelle distribution [116]. The chemical composition of PHAs determines their soluble nature in various solvents, mcl-PHAs are mostly soluble in a lot of solvents which include acetone and ethyl acetate. Another critical aspect of PHA pellet separation appears to be that the processes used must allow the polymers’ molecular size to change as little as possible.

### 6.1. Solvent Extraction

This was a popular technique because of its easy of usage with accessibility. Solvents induce the cellular surface to rupture, and increase diffusivity. Fluid interaction with PHA particles causes polymer solubilization. To recover the item, it should be precipitated with a non-solvent. Acetone and ethyl acetate are the solvents preferred for the separation of mcl-PHAs, whereas cold methanol or ethanol are acceptable non-solvents used for precipitation. The advantage of solvent separation is that it yields high-purity PHAs with nearly no change in particle size [117]. Many extraction methods use chloroform, although it is dangerous and increases the cost of the process. Endotoxin content is low in chloroform extracted PHAs, which is crucial for PHAs used in pharmaceutical applications. In several countries, chlorinated agents are no longer permitted in consumer goods. At room temperature, mcl-PHAs can be isolated using diethyl ether, tetrahydrofuran, or acetone [118]. Endotoxins (e.g., lipopolysaccharides) must be removed during PHA separation, especially if the product is to be used as a pharmaceutical. Thermally controlled separation procedures, as reported by Ferrur et al. (2007) for isolation of P(3HO-co-3HHx) from *Pseudomonas putida* GPo1, are one possible approach [119]. Organic solvents such as 2-propanol and n-hexane were used to achieve PHAs quality of more than 97% (*w*/*w*) and very less endotoxin limits (i.e., 10–15 endotoxin units per gram of PHO). PHO was also redissolved in 2-propanol at a temperature of 45 °C and precipitated at a temperature of 10 °C, providing a high purity product with low endotoxicity (2 per gram PHO). Green PHA isolation processes will necessitate less energy requirement, no harmful chemical usage, and excellent purity and yields [120]. Yabueng et al. (2018) examined green solvents such as 1,3-propanediol, 2-methyltetrahydrofuran, ethyl lactate, and 1,3-dioxolane for PHA separation from *Cupriavidus necator* [121].

### 6.2. Ultrasound-Assisted and Aqueous Two-Phase Extraction

Ishak et al. (2016) used ultra-high-frequency sound to irradiate mcl-PHAs with cells mixed with an excellent and minimal non-solute-solvent combination [122]. When that solvent is mixed in a sufficient proportion with a suitable solvent, the PHAs stay in suspension [123]. If the marginal solvent concentration is raised, the PHAs will be dissolved. These researchers evaluated impacts of ultrasonic irradiation maximal dissipation on energy, solvent/marginal non-solvent ratio, and time on mcl-PHA removal efficiencies. Furthermore, employing ultrasound during PHA extraction decreases solvent quantities, allowing safer solvents to be used, and it also shortens process times [124].

Aqueous two-phase extraction (ATPE) has the advantage of having a larger water concentration (up to 90% *w*/*w*), which makes biopolymer separation more ecologically friendly. Furthermore, the phase-separation components of ATPE can be harmless and relatively favorable. ATPE is said to be a scalable, cost-effective PHAs separation technique [9,125,126]. Leong et al. reexamined the parameters for purifying PHA from *C. necator* [127]. Additional centrifugation stages were avoided under these circumstances, and the recovery yield and purity factor increased by 72.2% and 1.61-fold, respectively [127]. As previously stated, non-PHA organic matter was removed to isolate PHAs from *C. necator* H16. The procedure was optimized using EOPO 3900 (5%), pH 6, and a fermenting temperature of 30 °C, purity factor and recovery yield were 1.36 and 97.6%, respectively [128].

### 6.3. Chemical and Enzymatic Digestion Method

By dissolving non-PHA cell material (NPCM), enzymatic and chemical digestion techniques aim to preserve unbroken PHA granules. The idea behind these methods is to break down microbial cell walls and release PHAs from the microorganism [129,130]. Early research looked towards removing NPCM from cells using powerful oxidizing agents such as sodium hydroxide and sodium hypochlorite. Extreme circumstances favorable for oxidation of both NPCM and PHAs—and therefore keen command of oxidizing agent content, heat generated, and reaction rate—is critical to this technique (i.e., reduced molecular weight). Dong et al. investigated surfactant and sodium hypochlorite mixtures for recovering PHAs from *Azotobacter cerococcid* G-3 [131]. Proteolytic enzymes catalyze hydrolytic processes involving proteins. According to preliminary research, lysozyme, bromelain, and trypsin are the most promising enzymes for this procedure. Yasotha et al. recovered and purified mcl-PHAs using alcalase, SDS, EDTA, and lysozyme [132]. Studies demonstrated that alcalase was the most important element in NPCM degradation and mcl-PHA purification. The counter flow filtration process successfully eliminated NPCM and allowed for high-purity mcl-PHA recovery [132]. PHA was purified by an enzyme method, according to Kachrimanidou et al. (2016) they developed a blend of unrefined enzyme to catalysis of *C. necator* lysis and release the PHAs compound using solid-state cultivation of *Aspergillus oryzae*. The enzymatic reaction was carried out at 48 °C with no control of pH. The product yield and quality of PHA achieved is 98% and 96.7%, respectively [133]. For the extraction of PHAs, Israni et al. took use of *Streptomyces albus* strong lytic activity [134]. *S. albus* and PHAs producing cultures are injected together into a reactor. The lytic enzymes from *S. albus* are added for PHA extraction in the second stage.

## 7. Properties of PHAs

One of the most significant characteristics for processing and producing polymers’ end-use is their thermal and physical qualities. The molecular structure of polymers is largely responsible for their physical and thermal characteristics. The physicochemical features of scl/mcl PHAs are distinct. Because PHAs are susceptible to increasing in temperature and breaking down during the melt process, their heat stability is essential. Plasticizers can help to prevent PHAs from degrading too much during the melting process [135]. Both P3HO and P3HO-P3HD have higher thermal degradation kinetic parameters than PHB and PHBV due to complexation on the development of six-membered transitional configuration. The thermal breakdown temperatures of P3HO with epoxy and hydroxy groups are varied. The P3HO with epoxy pendant groups might have a higher heat breakdown temperature after raising the temperature due to the bridging potential of epoxy groups.

### 7.1. Melting Point, Crystallization, Gas Barrier, and Solubility

Polymer performance is influenced by their crystallization kinetics. In PHAs, the length of the side chains can have a big impact on how they crystallize; for example, mcl-PHAs can form a layered crystal structure, increasing the side chain length of mcl-PHAs (e.g., C7 or more) and resulting in the formation of a new smectic liquid crystalline phase [136]. Both the main and side chains of mcl-PHAs with smectic crystals can cause cold crystallization during heating [32]; therefore, two melting points in mcl-PHAs have been found, which can be ascribed to the development of two distinct crystal phases (phase I and phase II) [137]. Crystallization rates have been shown to be improved by reactive chemical modification of PHAs. When compared to unmodified PHAs, modified PHAs with lengthy chain branching and low cross-linking densities demonstrated enhanced crystallization rates [138,139,140]. These chemical alterations also enhanced the rheological characteristics and processability of the material. Scl-PHAs require halogenated solvents to dissolve due to their high crystallinity [141]. The low crystallinity of mcl-PHAs permits them to be dissolved in non-halogenated solvents. The extraction, purification, and recycling of mcl-PHA copolymers are made easier and more cost-effective because of their solubility profile. Sobieski et al. (2017) reported the physical gel formation of P(3HB-co-3HHx) copolymer, and the gel’s thermal reversibility was established by heating it to 85 °C [142]. The composition of 3HHx in P(3HB-co-3HHx) might affect physical and mechanical strength, and rheological qualities in addition to polymer concentration. P(3HB-co-3HHx) gels with a low concentration of 3HHx (3.9 mol %) exhibit a greater opacity and better structure preservation when tilted than gels with a higher 3HHx content (13 mol %). After removing the solvent, the PHBHx gels revealed a sub-micron scale interconnected porous topology, indicating a viable material for biological applications such as tissue engineering [142].

When varying monomers with three carbon units, the melting point and crystallinity were reduced [143]. The quantity of side-chain carbons affects their melting and glass transition temperatures [144]. By copolymerizing P3HB with mcl-PHAs, the glass transition temperature of P3HB may be greatly decreased. The glass transition temperature of the mcl-PHAs generated from the C1–C7 side chain was between 0 °C and 50 °C, with a melting point of 175–69 °C [136]. Mcl-PHAs with slow crystallization rate and low melting point have limited melt processability; reactive modifications—including crosslinking, mixing with other polymers, and grafting techniques—can be used to solve these problems in the PHAs [145].

The oxygen and water vapor barrier qualities of polymers are important as their physical and thermal properties when it comes to using them for effective packaging applications such as food packing. When compared to traditional polymers, such as polypropylene and polyethylene, PHAs have better gas barrier qualities [146]. In particular, PHA copolymers have significantly better oxygen, carbon dioxide, and odor barrier characteristics than polyethylene and polypropylene [106]. PHBV has the best resistance to oxygen and water vapor penetration among biodegradable polymers [147]. When compared to PLA and PBS, the PHBV offers good potential for usage in fruit, and vegetable packaging as it has an appropriate oxygen and water vapor transfer rate [147]. However, the PHBV’s gas barrier qualities are insufficient for the packaging of meat, cheese, and coffee. Graphene oxide and clay supplementation to PHAs has been found to increase barrier characteristics by creating a convoluted route for gas molecules [148].

### 7.2. Mechanical Properties of PHAs

Because of their molecular weight and chemical makeup, the mechanical characteristics of PHAs can vary greatly. Tensile strength and Young’s modulus are 43 MPa and 3.5 GPa, respectively, for a typical P3HB with a molecular weight of less than 1000 Kda [149]. Because of secondary crystallization at room temperature, the brittleness of the P3HB was demonstrated to rise even more with age [150]. Copolymerization with other monomers can compensate for P3HB’s lack of toughness [149]. It was also shown that employing a modified *E. coli* strain to increase the molecular weight of the P3HB (2200 KDa) during synthesis might improve the mechanical qualities even further [151,152]. Molecular weight of the mcl-PHAs, varies from 45 to 462 Kda. Mw, Mn, and PDI of mcl-PHAs produced using various carbon sources and bacteria were summarized in Table 3. Mcl-PHAs are soft, flexible, sticky depending on the monomer composition. Copolymerization using as little as 5 mol % mcl monomers can improve the attributes of scl-PHAs (P3HB), such as toughness and thermal properties [143,153]. When P3HB was copolymerized with 17 mol % 3HHx, the elongation at break rose from 6% to 850%, while the P3HB’s tensile strength reduced from 43 to 20 MPa [153]. PHA copolymers with differing side group chain lengths or co-monomer compositions have variable modulus and elongation properties, as predicted.

## 8. Applications of PHAs

PHAs have shown considerable promise in various applications over the last two decades in the packaging, agricultural, and medical sectors in applications such as drug carriers, medical implants, and biocontrol agents [160]. The key criteria that govern the use of PHAs are their molecular weights and co-monomer concentration [143]. When compared to P3HB, P3HBV copolymer offers superior physical qualities such as impact resistance, hardness, flexibility, lower processing temperature, and a wider processing window [161]. Mcl-PHAs can be used as adhesives [162], coatings [163], plasticizers, medical devices, fibers [164], non-woven composites [165], and as bio-carriers in the slow release of fertilizers [166].

Crosslinking improves the mechanical qualities of PHAs, they can be employed in cartilage and ligament substitutes [167]. TEPHA Medical Devices Inc., based in the United States, has been manufacturing FDA-approved PHA sutures for medical absorbable sutures under the TephaFLEX^®^ brand since 2007 [168]. Unilever developed and launched PHA micro powder-based sun creams in 2019 to improve sun creams’ water resistance while lowering their environmental effect. This sun lotion is a natural alternative to microbead-based sunscreens, which can damage the seas and oceans. Danimer Scientific and Cove have produced entirely biodegradable straw and bottles made of PHAs. Nestle has also teamed up with Danimer Scientific to develop biodegradable water bottles, with the goal of making all plastic packaging recyclable or reusable by 2025 [169]. Overall, PHAs have applications in plasticizers, fishing lines [170], wastewater treatment [171], food packaging, and fertilizers [172].

Research on mcl-PHAs and their derivatives, which are more elastomeric, has intensified in recent years. Due to the limitations of large-scale production of mcl-PHAs, most recent studies have focused on the copolymers P(3HB-co-3HHx) and P(3HO), which have better mechanical characteristics and many more elastomers than scl-PHAs in addition to having been manufactured in considerable amounts. Because many organs in the body have elastomeric qualities, mcl-PHA polymer scaffolds that can withstand and retake from numerous structures without harming other nearby tissue can be used. Tim et al. employed a polymeric scaffold made up of two components fashioned into a tubular conduit, the inner layer was made using a polyglycollic acid mesh and the outer layer was made with three layers of non-porous P(3HO-co-3HHx) with 10% 3HHx for tissue engineering studies [172].

P(3HB-co-3HHx) scaffolds can thus be employed for vascular grafting, according to these findings [173]. Sodian et al. conducted one of the first investigations employing an elastomeric P(3HO) for the creation of a tri-leaflet heart valve scaffold [174]. Based on the findings of these experiments, it was shown that P(3HO) manufactured TEHVs may be implanted in the pulmonary location and operate properly for 120 days in lambs [174]. Stock et al. (2000) investigated the possibility of developing three-leaflet, valved pulmonary conduits in lambs using autologous ovine vascular cells and thermoplastic P(3HO) [175]. Sodian et al. (2002) employed a new stereolithography manufacturing approach to create P(4HB) and P(3HO) scaffolds of cuspid and semilunar valves based on X-ray computed tomography and appropriate software [176]. Wu et al. (2007) created unique hybrid valves made of decellularized swine aortic valves covered with PVC (3HB-co-3HHx) [177]. Yang et al. (2002) conducted one of the first experiments on P(3HB-co-3HHx) as a nerve regeneration conduit material [178]. Ying et al. (2008) used electro spun copolymers of P(3HB-co-5 mol % 3HHx), P(3HB-co-7 mol % 4HB) and P(3HB-co-97 mol % 4HB) to make nanostructured fibrous scaffolds [179]. The generated scaffolds have tensile strength and Young’s modulus in the ranges of 5–30 and 15–150 MPa, which are equivalent to human skin. P3HO homopolymer fabricated 2D films were also investigated as a matrix material for skin tissue engineering. Wang et al. (2004) tested the in vitro biocompatibility of rabbit bone marrow cells injected on PLA, P3HB, and P(3HB-co-3HHx) 3D scaffolds [180]. On the P(3HB-co-3HHx) scaffolds, the cells proliferated the most. Several studies on three-dimensional polymer scaffold systems using a blend of P(3HB) and P(3HB-co-3HHx) for use as a matrix for cartilage tissue engineering have been conducted [181,182,183]. Wang et al. conducted one of the things is to first investigations of the use of PHA with dendrimer matrix for effective skin drug delivery systems (SDS) [184]. Vermeer et al. (2021) demonstrated a new application of PHAs in self-healing concrete [185]. Harazna et al. (2020) used ceramic-polymer bounded diclofenac with the biocompatible P(3HO) [186].

## 9. Conclusions and Future Perspectives

This review summarized the recent developments in mcl-PHA production using organic/inorganic carbon sources and various types of the fermentation strategies such as batch, continuous, and fed-batch. We also discussed downstream processing methods, properties and applications of mcl-PHAs, and their worldwide production at an industrial scale. Current mcl-PHA production through fermentation is facing several problems, such as high levels of foaming in the reactor due to toxicity of fatty acids which are used as a carbon source, inconsistency in the PHA titers (varying from 3 to 102 g/L), polymer property alterations due to fluctuations in molecular weights (from 45 to 462 Kda of Mw). Fatty acid (high) concentration in the fermentation broth inhibiting the growth of microorganisms and creating problems in down-stream processing, such as centrifugation and lyophilization. High-cell-density fermentation requires a continuous oxygen supply (7 L/min) which is expensive and creates safety issues in large-scale fermentation. Before scaling-up the mcl-PHA production process, researchers should focus on solving the above-mentioned problems by using recombinant strains and wild type bacteria other than *Pseudomonas*.

## Figures and Tables

**Table 1:** Mcl-PHA production using various carbon sources from literature reports.

Bacteria	Carbon Source	DCW (g/L)	PHA Conc. (g/L)	PHA (%)	PHA Composition	Productivity (g/L/h)	Time (h)	References
*P. putida* KT2442	Oleic acid	141	72	51	C6-C8-C10-C12-C14	1.91	38	[10]
*P. putida* KT2440	Glucose + nonanoic acid	98	32	33	C9-C7	3.1	32	[13]
Recombinant *R. eutropha*	Palm oil	139	102	74	Mcl-PHA	1.2	96	[27]
*P. putida* KT2440	Waste cooking oil	159	58	36	C6-C8-C10-C12-C14	1.93	30	[28]
*P. putida* KT2440	Glucose + nonanoic acid + acrylic acid	71	53	75	C9-C7	1.8	30	[29]
*P. putida* LS46	Octanoic acid	29	17	61	C6-C8-C10-C12	0.66	27	[30]
*P. putida* KT2440	Glucose + nonanoic acid	71	40	56	C5-C7-C9	1.44	28	[31]
*P. putida* IPT 046	Glucose + fructose	50	31	63	Mcl-PHA	0.8	42	[32]
*P. oleovorans*	n-octane	112	5.6	5	Mcl-PHA	0.091	61	[33]
*P. putida* KT2442	Octanoic acid	51	18	35	C8	0.41	43	[34]
*P. putida* KT2442	Oleic acid	90	18	20	C6-C8-C10-C12-C14	0.57	32	[34]
*P. oleovorans*	n-octane	12	3.4	28	Mcl-PHA	0.58	120	[35]
*P. oleovorans*	n-octane	37	12	33	Mcl-PHA	0.25	48	[36]

*P.* = *Pseudomonas*; DCW = Dry cell weight; PHA conc. = polyhydroxyalkanoates concentration; *R.* = *Ralstonia*.

**Table 2:** Mcl-PHA production at various modes of operations from literature reports.

Bacteria	Substrate	Mode of Operation	PHA Production	Reference
*P. putida KT2440*	Glucose and nonanoic acid	Fed-batch mode	32%	[13]
*P. mendocina*	Octanoate	Batch mode	31%	[82]
*P. oleovorans*	n-octane	Continuous mode	63%	[83]
*P. putida*	Oleic acid	Fed-batch mode	51%	[84]
*P. putida LS46*	Octanoic acid	Fed-batch mode	61%	[30]
*P. putida KT2440*	Decanoic and acetic acids	Fed-batch mode	74%	[85]
*P. putida KT2440*	Glucose and nonanoic acid	Fed-batch mode	56%	[31]
*P. putida CA-3*	Decanoic and butyric acid	Fed-batch mode	65%	[86]
*P. putida KT2440*	Nonanoic acid	Fed-batch mode	75%	[87]
*P. oleovorans*	Octanoic acid	Fed-batch mode	62%	[88]
*P. putida BM01*	Glucose and octanoate	Fed-batch mode	66%	[89]

**Table 3:** Gel permeation chromatography (GPC) results of mcl-PHAs from literature reports.

Bacteria	Carbon Source	Mw (kDa)	Mn (kDa)	PDI	References
*P. putida* KT2440	Waste cooking oil	45	22	2.04	[28]
*P. putida* KT2440	Nonanoic acid + undecanoic acid	115	-	1.8	[31]
*P. putida* KT2442	Oleic acid	135	49	2.76	[34]
*P. putida* KT2442	Octanoic acid	187	78	2.4	[34]
*P. putida* KT2442	Vegetable-free fatty acids	168	65	2.68	[34]
*P. putida* KT2442	Animal-free fatty acids	180	71	2.53	[34]
*P. putida* KT2440	Biodiesel-derived crude glycerol	462	193	2.4	[154]
*P. putida* LS46	Hexanoic acid	49	22	2.3	[155]
*P. putida* LS46	Heptanoic acid	82	35	2.3	[155]
*P. putida* LS46	Octanoic acid	115	54	2.2	[155]
*P. putida* LS46	Nonanoic acid	55	26	2.3	[155]
*P. putida* LS46	Decanoic acid	49	21	2.4	[155]
*P. putida* LS46	Lauric acid	131	63	2.1	[155]
*P. putida* LS46	Myristic acid	86	44	2.0	[155]
*P. putida* KT2442 mutant	Dodecanoic acid (15%)	100	80	1.25	[156]
*P. putida* KT2442 mutant	Dodecanoic acid (39%)	157	108	1.45	[156]
*P. putida* KT2442 mutant	Tetradecanoic acid (49%)	95	67	1.43	[156]
*P. corrugat*	Coconut oil	343	74	4.6	[157]
*P. resinovorans*	Coconut oil	165	101	1.63	[158]
*P. resinovorans*	Sunflower oil	112	65	1.72	[158]
*P. resinovorans*	Soybean oil	127	70	1.81	[158]
*P. oleovorans*	Octanoic acid	189	51	3.69	[159]
*P. oleovorans*	Soybean oil	130	72	1.70	[159]
*P. oleovorans*	Undecanoic acid	260	135	1.92	[159]

*P.* = pseudomonas; Mw = molecular weight; Mn = molecular number; PDI = polydispersity index.

## Data Availability

Data is contained within the article or corresponding author.
